# Digital technologies for monitoring infected people, identifying contacts and tracking transmission chains in the corona virus disease 2019 pandemic: A protocol for a systematic review

**DOI:** 10.1097/MD.0000000000023744

**Published:** 2020-12-18

**Authors:** Talita Araujo de Souza, Arthur de Almeida Medeiros, Isabelle Ribeiro Barbosa, Gilson de Vasconcelos Torres

**Affiliations:** a Postgraduate Program in Health Sciences; b Integrated Health Institute, Federal University of Mato Grosso do Sul, Campo Grande/MS, Brazil; c Postgraduate Program in Public Health, Federal University of Rio Grande do Norte, Natal/RN, Brazil.

**Keywords:** corona virus disease 2019, digital technology, disease transmission, epidemiological monitoring, public health surveillance

## Abstract

**Background:**

In times of the corona virus disease 2019 (COVID-19) pandemic, due to the urgent need to respond quickly to the challenges posed by the introduction of a new etiological agent and the peculiarity of the disease, which poses risks to people's lives and health, the use of digital technologies for monitoring and surveillance have been used as a means of fighting coronavirus. Thus, this study will identify the use of digital technologies to monitor, identify contacts and track transmission chains of COVID-19 worldwide.

**Methods:**

The systematic review of this protocol will follow the guidelines of the Preferred Reporting Items for Systematic Reviews and Meta-Analyzes Protocols. We will include studies that present digital technologies used in the monitoring of infected people, contact identification and the transmission chain of COVID-19 developed worldwide. For the selection of articles, the following databases will be consulted: PubMed, EMBASE, LILACS, Web of Science, Science Direct, Scopus, Livivo and CINAHL. In addition, we will conduct extensive research on selected sources of gray literature, including bibliographic databases, web-based search engines, practice-oriented magazines and government websites. Data extraction will take place in 2 stages (1- title and abstract screening and 2- full-text screening) and will be carried out independently by 2 reviewers, using the Mendeley software and the Rayyan QCRI application. The studies will be characterized as to the type and design of the study in relation to the ease in demonstrating the technologies used and the type of information produced. If it is necessary to synthesize quantitative data, the heterogeneity assessment will be performed using I2 statistics, and the meta-analysis will be processed using Review Manager 5.3.

**Results:**

The development of this research will allow the knowledge of how these technologies were applied according to each territory and their effectiveness in reducing cases of COVID-19.

**Conclusion:**

The results of this review can reveal the importance of modern technologies for reducing cases of COVID-19 and that these measures can be adopted by governments, organizations and for everyone

**Record of systematic review:**

CRD42020211744.

## 1 Introduction

In December 2019, an outbreak of contamination by a new zoonotic agent, later identified as Severe Acute Respiratory Syndrome Coronavirus 2 (SARS-CoV-2), was observed in Hubei province in China, and the disease as a result of this new coronavirus was called of corona virus disease 2019 (COVID-19), and since then the disease has reached more than 200 countries and territories spread all over the world, with more than 36 million confirmed cases and more than 1 million deaths.^[1–3]^

As it is a new disease, many issues are not fully clarified and situations that pose challenges for monitoring the course of the pandemic are also identified, such as reduced laboratory testing capacity, frequent delays in disseminating results, variability in the course of the disease in different territories and the lack of clarity in communication in some nations. Such situations have hampered the development of policies and interventions focused on resource allocation. At a time when conventional health surveillance capabilities are limited, electronic social media is an important strategy for identifying an emerging outbreak.^[4]^

In this scenario, research in surveillance, called infovigilance or infodemiology, presents itself as a strong expectation in the use of internet data to track the development of health problems in real time, with greater attention and more precise time.^[5–8]^

The growing production and use of data based on increasingly powerful and specialized digital technologies has enabled the emergence of new forms of knowledge production based on sophisticated computational modeling and algorithms. In this new context, data gains value and importance through diverse mobilizations that involve negotiations, social, political and economic interests. In times of COVID-19 pandemic, due to the urgent need to respond quickly to the challenges posed by the introduction of a new etiological agent and the peculiarity of the disease, which poses risks to people's lives and health, the use of personal data from different sources being required to explore scientific issues based on the characteristics of the population, laboratory and hospital data, among others, as long as it is guided by an ethical and legal basis.^[9]^

The use of data-based monitoring and surveillance applications has been used as a means of combating coronavirus. Use of technologies based on facial recognition, installation of thermometers at airports, applications that track where infected people have walked are examples of some applications of digital surveillance in combating the pandemic.^[10]^

So, this study will identify the use of digital technologies to monitor, identify contacts and track transmission chains from COVID-19 worldwide.

## 2 Methods and analysis

### 2.1 Protocol and registration

This systematic review was recorded in the International prospective register of Systematic reviews (PROSPERO) on Sep 14, 2020 under the number CRD42020211744. Available at: https://www.crd.york.ac.uk/prospero/display_record.php?ID=CRD42020211744.

### 2.2 Selection process

**Figure 1:**
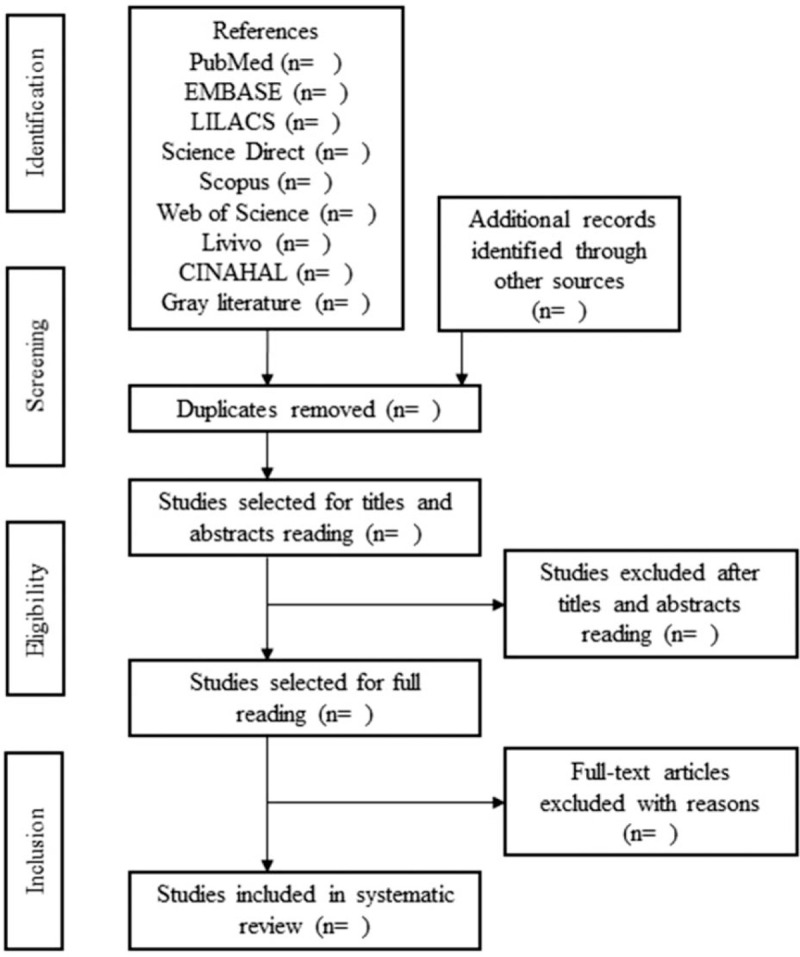
Flow diagram. Adapted from PRISMA-P. PRISMA-P = preferred reporting items for systematic reviews and meta-analyses protocols.

The design and development of this systematic review and meta-analysis will be in accordance with the statement of Preferred Reporting Items for Systematic Reviews and Meta-Analyses.^[11]^ In this systematic review, the following databases will be considered: PubMed, EMBASE, LILACS, Web of Science, Science Direct, Scopus, Livivo and Cinahl. We will undertake extensive searches of selected grey literature sources including bibliographic databases, web-based search engines, practice-orientated magazines and governmental websites. In order to carry out the appropriate search in each database, the search strategy will be duly modified for each 1 and will be carried out by 2 reviewers in a double-blind manner to identify the eligible studies. This pair of independent researchers will carry out the search, and publications considered to be potentially relevant will be included in the review if they meet all the inclusion criteria. Consensus meetings will be held at each stage, if there is no consensus the third reviewer will participate. The reference list of possible studies included will be selected manually to identify other relevant publications. In case of disagreement, it will be resolved by a third reviewer. Figure 1.

### 2.3 Search strategy

As keywords for search strategy will be considered: Technology; digital technology; Mass Screening; Mobile Applications; Epidemiological Monitoring; Public Health Surveillance; Disease Transmission, Infectious; COVID-19; Coronavirus Infections; Humans; Pandemics.

The keywords search will be based on Medical Subject Headings and the search strategy described will be adapted and customized for each database to be searched using Boolean operators, truncations and proximity operators, as appropriate.

### 2.4 Inclusion criteria

Studies that report the use of digital technologies to monitor suspected and confirmed cases and to track transmission chains of COVID-19 will be included, regardless of the type of study, the type of institution that proposes/executes the tool (government institutions, government agencies, government agencies or research institutions), language and place of use. Table 1.

**Table 1:** PICO description.

PICO	Abbreviation	Elements
Participants	P	Digital Technologies
Exposition	E	Identification of cases and suspects and tracking of transmission chains of COVID-19.
Control	C	No control needed
Outcome	O	Technologies for monitoring suspected and confirmed cases of COVID-19.

### 2.5 Exclusion criteria

We will exclude studies that do not report the use of digital technologies to monitor suspected and confirmed cases and to track transmission chains of COVID-19.

### 2.6 Data collection process

Data extraction will be taken independently by 2 reviewers in 2 stages: Title and abstract screening and full-text screening.

The studies will be imported by Mendeley software^[12]^ and duplicate articles will be excluded. After that, the selected articles will be exported to the Rayyan QCRI application^[13]^ for evaluation of title and abstract. The reasons why the articles were excluded will be described by the 2 reviewers.

In the second stage, the full-texts of the studies selected in the previous stage will be analyzed.

If any disagreement remains on eligibility, it will be discussed between the first 2 reviewers and the third review. Disagreements will be resolved by discussion in all stages.

Final selection will be always based on the full-text of the publication. Excluded full-text studies will be listed with the reason for their exclusion, and a preferred reporting items for systematic reviews and meta-analyses flow-diagram of included studies will be produced.

Two independent reviewers (1R and 2R) will collect data from the selected articles. After this stage, they will crosscheck the retrieved information. If there are any disagreement will be discussed between them and the third reviewer. If necessary, the expert becomes involved to make a final decision.

The data will be extracted using a standardized data-extraction form created a priori.

The information to be extracted will be:

- Study information: Title, author(s), year, journal, location; geographic setting, inpatient/outpatient
- Study design and duration
- Study participants: number of participants, sample characteristics (age, gender)
- Phenomenon of interest: description of the specifics of the monitoring tools; Type of technology used
- Comparators: details of control conditions (not mandatory)
- Evaluation: Different interventions, outcomes, effectiveness/importance will be assessed by evaluating the majority of study's findings.

### 2.7 Risk of bias assessment

There is no immediate need to individually assess the quality of each study. The studies will be characterized according to their type and study design in relation to the ease in demonstrating the technologies used and the type of information produced.

Selection bias and publication bias will be minimized by using independent validation of coding decisions and by comprehensive research from unpublished sources in journals.

### 2.8 Data synthesis

If quantitative synthesis is appropriate, a method of meta-analysis will be performed using Review Manager 5.3 (RevMan 5.3, The Nordic Cochrane Centre, Copenhagen, Denmark). Heterogeneity will be calculated by inconsistency indexes (I-squared), and a value greater than 50% was considered an indicator of substantial heterogeneity between studies. The significance level was set at 5.0%.^[14]^

### 2.9 Confidence in cumulative evidence

The grading of recommendations, assessment, development, and evaluation approach will be used to assess the quality of evidence that will be included in this review

### 2.10 Ethics and dissemination

As it is a systematic review protocol and there will be no patient participation, it is not necessary to conduct an ethical review of the study. The results will be presented at congresses and published in a peer-review journal. The results of this review may support the development and improvement of digital technologies that enable the monitoring of COVID-19 and assist in the reduction.

## 3 Discussion

This study will be the first study to compile results about the development, use of digital technologies for the monitoring and screening of COVID-19. Its development will allow knowledge of how these technologies were applied according to each territory and their effectiveness in reducing cases of COVID-19. We hope that the results of this review can reveal the importance of modern technologies for reducing cases of COVID-19 and that these measures can be adopted by governments, organizations, and for everyone.

## Author contributions

**Conceptualization:** Talita Araujo de Souza, Arthur de Almeida Medeiros, Isabelle Ribeiro Barbosa.

**Data curation:** Talita Araujo de Souza.

**Formal analysis:** Arthur de Almeida Medeiros, Isabelle Ribeiro Barbosa.

**Funding acquisition:** Arthur de Almeida Medeiros, Gilson de Vasconcelos Torres.

**Investigation:** Talita Araujo de Souza, Isabelle Ribeiro Barbosa.

**Methodology:** Talita Araujo de Souza, Arthur de Almeida Medeiros, Isabelle Ribeiro Barbosa, Gilson de Vasconcelos Torres.

**Project administration:** Arthur de Almeida Medeiros, Isabelle Ribeiro Barbosa, Gilson de Vasconcelos Torres.

**Supervision:** Isabelle Ribeiro Barbosa, Gilson de Vasconcelos Torres.

**Validation:** Talita Araujo de Souza.

**Writing – original draft:** Talita Araujo de Souza.

**Writing – review and editing:** Talita Araujo de Souza, Arthur de Almeida Medeiros, Isabelle Ribeiro Barbosa, Gilson de Vasconcelos Torres.
